# Evaluation of a brief video intervention aimed at UK-based veterinary surgeons to encourage neutering cats at four months old: A randomised controlled trial

**DOI:** 10.1371/journal.pone.0263353

**Published:** 2022-02-09

**Authors:** Jenni McDonald, Jane Clements

**Affiliations:** 1 Veterinary Department, Cats Protection, National Cat Centre, Haywards Heath, United Kingdom; 2 Bristol Veterinary School, University of Bristol, Bristol, United Kingdom; University of Lincoln, UNITED KINGDOM

## Abstract

In the UK, it is currently recommended that owned cats be neutered from four months of age. However, its uptake is inconsistent across the veterinary profession. Here we assess the effect of a brief video intervention that aimed to encourage four month neutering, whilst preserving clinical autonomy. We compare this theory-driven approach with traditional information giving and a control group. Veterinary surgeons who regularly undertook feline neutering work in the UK but did not routinely neuter cats at four months and/or recommend four month neutering for client owned cats were randomised into three groups (n = 234). Participants received either no information, a written summary of evidence or the video. The primary behaviour outcomes were the recommending and carrying out of neutering cats at four months. Evaluative, belief and stages of change measures were also collected. Self-reported outcomes were assessed pre-intervention, immediately post-intervention, two months post-intervention and six months post-intervention. At two months, participants that had received the video intervention were significantly more likely to have started recommending neutering cats at four months. At six months, participants that had received the video intervention were significantly more likely to have started carrying out neutering cats at four months. There were no significant behaviour changes for the other groups. At two months, the video intervention was associated with a significant increase in thinking about, and speaking to colleagues about, four-month neutering, relative to the control group. The written summary of evidence had no similar effect on stages of change, despite it being perceived as a significantly more helpful resource relative to the video. To conclude, a brief one-off video intervention resulted in an increase in positive behaviours towards neutering cats at 4 months, likely mediated by the social influences of the intervention prompting the opportunity to reflect and discuss four-month neutering with colleagues.

## Introduction

Globally, the number of unowned cats is a cause for concern [1–4]. In the UK, shelters are often at capacity [5,6] with many cats on rescue centre waiting lists [6], and stray and feral cats commonly found in large numbers across localised areas [7]. A key effort to address this is the prevention of accidental litters in the owned cat population. Despite high neutering rates of owned cats in the UK (86–88% per cent in 2020; [8,9]), neutering has to be carried out early enough to prevent accidental litters. The traditional age of neutering was six months, however cats can reach puberty at four months of age [10]. Since 2006 neutering owned cats prepubertally from four months of age has been recommended, and is currently supported by national animal welfare organisations [11,12] and endorsed by national veterinary associations [13,14]. Despite a general trend towards earlier surgery [15,16], only half of veterinary surgeons based in the UK are in line with recommendations, routinely recommending neutering at four months old for client-owned cats [8,15].

The majority of owned cats are not neutered before a possible age of breeding [17], with a high proportion of litters accidental [18]. Although, there can be many barriers to four-month neutering and neutering generally [19], one key barrier may be due to the recommendations of veterinary professionals [20]. Despite, approximately 70 per cent of veterinary surgeons indicating they are comfortable carrying out neutering on cats of four months of age many consider it context specific and not applicable to the owned cat population [15,21]. Additionally, veterinary surgeons make decisions often in time- and resource-pressured environments where decision-making may become subject to routines and habits, which is likely to be the case with regard to neutering cats, given six months was the traditional recommended age of neutering. Although the UK has a significantly higher average age of neutering compared to Australia and New Zealand [21], where prepubertal neutering is more common place, studies suggest similar disparities in attitudes and behaviours towards prepubertal neutering occur within the veterinary profession elsewhere (e.g. Australia [21,22], France [23], New Zealand [21], USA [24]). Indeed, even in areas where prepubertal neutering is a legal requirement, not all veterinary surgeons make the recommendation to their clients [25]. Consequently, there is a need to use this understanding for effective development of interventions, to increase recommendation of the neutering procedure at four months, to make this the social norm and routine practice, thereby reducing the numbers of accidental litters being born. Recent results suggest barriers to carrying out and recommending neutering for client-owned cats tend to be around three broad themes; (1) Informational needs around the health consequences of neutering cats at four-months; (2) Training needs around the appropriate protocols and procedures to neuter cats at four-months; (3) Social influences, including peers and cat owners and perceptions of social norms [15].

To date, approaches have largely focussed on the informational needs, such as educational materials, written evidence and lectures. Indeed, the concerns of the veterinary profession in relation to the perceived negative consequences of prepubertal neutering are addressed in many studies that to date find no evidence for increased risks [e.g. 26–29]. These educational approaches are reflected in an increase in uptake over the past decade [15,21,30]. Additionally, training resources and protocols are available online [12]. However, provision of educational behaviour change techniques in the form of information giving alone has been demonstrated to be ineffective within health behaviour research [31], this may go some way to explain why the earlier neutering message has so far failed to engage and change the behaviour of some veterinary professionals. An additional consideration is the importance of within-practice experience and social norms resulting in contrasting belief “silos” within the profession [15,23], which potentially act as a barrier to further uptake. Consequently, as advocated generally when developing behaviour change interventions, an evidenced theory-driven approach is required that addresses the factors that influence four-month neutering. Here we detail the development and evaluation of an intervention based on a behavioural analysis using the COM-B (capability, opportunity, motivation-behaviour) model and behaviour change wheel (BCW) [32].

## Intervention design

The COM-B system provides a simple model to explain the basic interacting elements of behaviour change. The core components of COM-B are physical capability (skills or strength) and psychological capability (knowledge and understanding), physical opportunity (environment) and social opportunity (availability of ideas and communication), and automatic and reflective motivation [32]. Automatic motivation drives behaviour through emotional impulses and habits, whereas behavioural outcomes achieved by evaluative thought processes would be due to reflective motivation. Analysing the components of COM-B in relation to the desired behaviour enables identification of the factors that influence change [33]. Knowledge and understanding of the barriers and belief systems underpinning current behaviour are vital to the successful development of interventions that will be most likely to influence the desired change, the Theoretical Domains Framework (TDF) provides a mechanism for doing this [34].

The key themes arising from research exploring the perceptions, perceived norms and familiarity and experience of veterinary surgeons regarding pre-pubertal neutering [15], allowed mapping of the evident barriers to the TDF, identification of the relevant components of COM-B and those of the BCW in order to design an appropriate intervention (Table 1). Four themes were identified as influencing behaviour towards neutering cats at four months and guided intervention design: (1) “Information about health consequences” (TDF domains included knowledge and beliefs about consequences); (2) “Information about others approval” (social influences, professional/social role and emotion); (3) “Demonstration of the behaviour” (social influences, professional/social role); (4) “Credible source” (social influences).

**Table 1:** The analysis of the elements of COM-B and the BCW to show how the video intervention relates to the framework.

Behaviour change technique	Relevant component of COM-B	Intervention functions	TDF	Video intervention
Information about health consequences	Psychological capability, and reflective motivation	Education and persuasion	Knowledge Beliefs about consequences	The benefits of four month neutering with regard to the kittens welfare and wellbeing due to shorter and easier surgery, shorter anaesthetic times, quicker recoveries and prevention of accidental litters are discussed.
Information about others approval	Psychological capability, automatic motivation and reflective motivation	Education and persuasion	Social influences Professional/social role Emotion	Discussion covered the numbers of veterinary surgeons already recommending neutering at four months, affirming that there is much approval for the behaviour within the profession. Many veterinary surgeons will not be aware of the relatively high number already making the recommendation
Demonstration of the behaviour	Social opportunity Reflective motivation	Modelling	Social influences Professional/social role	Raising the subject with kitten owners and an example conversation. Providing an observable example to imitate.
Credible source	Reflective motivation	Persuasion	Social influences	Communication given by a high status, well-respected authority who emphasises the importance of four month neutering for the welfare of cats.

Consequently, our intervention consisted of a video of a well-respected authority in feline medicine, specifically a recognised Royal College of Veterinary Surgeons Specialist in Feline Medicine, discussing the benefits of neutering at four months and strategies for recommending this to their clients, via a conversation with a colleague. The video-format suits the time-pressured environments of veterinary surgeons and the content set out to provide an opportunity for practitioners to reflect on their neutering practises, which may have become subject to personal habits given the historical recommendation of six months. Specifically the video provided information about how other veterinary surgeons behave, how cat owners perceive four month neutering and the reasons and benefits to cat welfare of starting neutering cats at a younger age, with the aim to change well-established habits. One key difference between this intervention and previous educational resources around four-month neutering is the recognition of the importance of social norms. Social norms are highlighted as important within both the veterinary profession and other health-professional scenarios, especially in situations where beliefs are contrasting. Indeed, veterinary surgeons that do not undertake four-month neutering are less likely to consider it widespread within the veterinary community generally [15]. Highlighting positive social norms by providing information about how most people behave is shown to be effective at changing behaviour [35,36]. This can also be linked to professional and social role, which demonstrates the power of social influence. Where there is uncertainty about undertaking a new practice, authority can play an important role [37], particularly if the communicator is also perceived as a trustworthy source, with authority and trust adding credibility to the message as persuasive factors [38]. Discussions by opinion leaders, as opposed to educational materials, have been found to be more effective at influencing physicians decisions [39].

Whilst, COM-B and the associated behaviour change wheel provided a ‘user-friendly’ and comprehensive model [32] that guided our intervention development, other theories are also relevant and were considered in the design of this intervention. Specifically, the social influences, knowledge and beliefs about consequences point to constructs within the theory of planned behaviour (TPB) including attitudes, subjective norms (approval by people important to you) and perceived control over behaviour [40]. Additionally, Bandura’s social cognitive theory (SCT) states that self-efficacy and outcome expectations (perception of positive outcomes from changing behaviour) will all be enablers of motivation to change [41]. This is evidenced by a study that saw more doctors’ recommending services following a SCT based intervention [42]. Similarly, the intervention designed for this study, addresses why neutering at four months has good welfare outcomes (Table 1); SCT would suggest this is the right intervention.

In addition to a control group, that received no intervention, we included another treatment group that received a written summary of evidence that provided a more traditional educational resource with regard to the health outcomes for cats and provided scientific references. We tested the efficacy of the educational materials and video intervention at influencing views, beliefs and behaviour change towards 4-month neutering.

## Methods

### Study design

**Fig 1:**
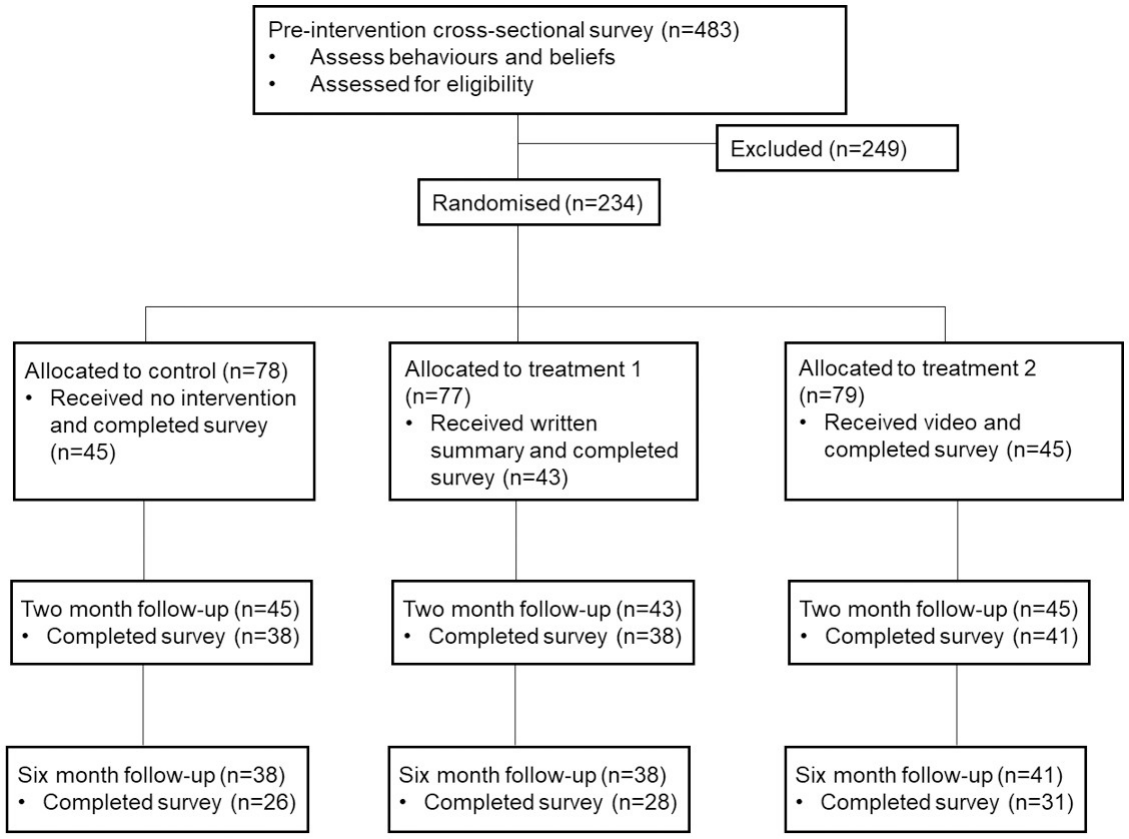
Study design and participant flow diagram.

The design was a three-by-four mixed design (Fig 1): Condition (treatment 1/treatment 2/control) x Time (pre-intervention/immediately post intervention/2-month follow up/6-month follow-up). The participants were veterinarians who regularly undertook feline neutering work but did not routinely neuter cats at four months and/or recommend four month neutering for client owned cats. Participants were chosen following a preliminary assessment of behaviours around cat neutering (see [15] for further details). Stratified by sex due to its known association with behaviours towards neutering within this study population [15] and whether they undertake or recommend neutering, participants were randomly assigned to control or treatment conditions using the block_ra () function in the randomizer package in R [43].

Group assignment included:

Treatment 1: Received a written summary of evidence
Treatment 2: Received a video intervention
Control group: Received no information

Respondents in the treatment groups had to confirm they had accessed and read or watched the content before continuing the survey. The final sample comprised 234 veterinary surgeons, 79% were female (n = 184).

Four broad groups of data were collected:

- Evaluative information was collected immediately following the intervention, relevant for treatment groups only, to provide immediate feedback on the intervention
- Likert-scale beliefs towards four-month neutering were collected pre-intervention and at a two- and six-month follow-up.
- Information on stages of change in the form of Likert scale questioning were included at 2 and 6 months and focussed on the months since the last survey, i.e. 2 and 4 months respectively. Stages of change was defined as an increased willingness with regard to four-month neutering in the following areas; thinking, openness to discussions, open to resources, resource-seeking, speaking with colleagues, speaking with cat owners and actively looking up protocols.
- Self-reported behaviour with regard to neutering cats at four-months and recommending four-month neutering for client owned cats were also collected.

The protocol was approved by University of Bristol’s Faculty of Health Science Research Ethics Committee (approval number 76 301).

### Statistical analysis

Ordinal logistic regressions were used using the polr() function in the MASS package [44] in the statistical programme R [43] to assess differences between treatment and control groups in terms of the agreeability of veterinary surgeons to Likert scales measures of intervention evaluation, stages of change and beliefs (S1 Dataset). We carried out analysis of variance on the variables, using likelihood ratio chi square tests to determine significance (included where *p* < 0.05) using the Anova() function in the car package [45] in R. For the beliefs measures we also included a time*treatment interaction term as the measure was repeated through time, whereas stages of change focussed on the months between surveys and evaluative measures were immediately post-intervention only.

We looked at the significance of any behaviour changes within each group, using McNemar chi-test that recognises that veterinarians may start and stop behaviours randomly, to determine whether there is a significant uptake in behaviours. We considered two separate time points for analysis (1) Pre-intervention vs. 2-month follow-up. (2) Pre-intervention vs. 6-month follow-up.

## Results

### Immediately following the intervention

The two treatment groups answered evaluative questions regarding the written summary of evidence and the video, to provide their initial feedback on the resource. Both groups were equally likely to agree that the intervention was relevant to them. However, veterinary surgeons that received the video intervention were three times more likely to disagree that the content was related to the knowledge they needed (OR 3.14, Table 2) and almost four times more likely to disagree the content provided a helpful resource (OR 3.91, Table 2).

**Table 2:** Agreement with evaluative statements regarding the received treatment.

Question	Group	N	Disagree	Neutral	Agree	Ordinal regression statistics
The content was relevant to me	Treatment 1	43	2 (4.7%)	4 (9.3%)	37 (86%)	LRT = 0.07, p = 0.81
	Treatment 2	45	3 (6.7%)	4 (8.9%)	38 (84.4%)	
The content was related to the knowledge I needed	Treatment 1	43	3 (7%)	5 (11.6%)	35 (81.4%)	LRT = 5.73, p = 0.02*
	Treatment 2	43	8 (18.6%)	10 (23.3%)	25 (58.1%)	
The content provides a helpful resource	Treatment 1	43	4 (9.3%)	2 (4.7%)	37 (86%)	LRT = 7.46, p = 0.006*
	Treatment 2	45	5 (11.1%)	14 (31.1%)	26 (57.8%)	
If freely available, I would recommend the summary of evidence/video to colleagues	Treatment 1	43	3 (7%)	4 (9.3%)	36 (83.7%)	LRT = 2.45, p = 0.12
	Treatment 2	45	4 (8.9%)	10 (22.2%)	31 (68.9%)	
The content provided useful information that will help me communicate with cat owners	Treatment 1	42	4 (9.5%)	3 (7.1%)	35 (83.3%)	LRT = 0.70, p = 0.40
	Treatment 2	45	5 (11.1%)	6 (13.3%)	34 (75.6%)	
The content provided useful information that will help me communicate with colleagues	Treatment 1	43	5 (11.6%)	7 (16.3%)	31 (72.1%)	LRT = 2.44, p = 0.12
	Treatment 2	45	8 (17.8%)	12 (26.7%)	25 (55.5%)	
The content made me think about my own neutering practises	Treatment 1	43	4 (9.3%)	11 (25.6%)	28 (65.1%)	LRT = 0.11, P = 0.74
	Treatment 2	44	6 (13.6%)	7 (15.9%)	31 (70.5%)	
The content highlighted the benefits of four month neutering	Treatment 1	43	5 (11.6%)	1 (2.3%)	37 (86.1%)	LRT = 0.92, p = 0.34
	Treatment 2	45	7 (15.5%)	3 (6.7%)	35 (77.8%)	

*indicates significant difference between veterinary surgeons in the treatment 1 and treatment 2 group.

### Two month follow up

Only respondents that completed the initial and the two-month follow up survey were included (n = 117, Fig 1).

At two months none of the veterinary surgeons in the treatment 2 group had accessed the video again (n = 0/41), whereas 13% of people who were in the treatment 1 group had accessed and read the summary of evidence document again (n = 5/38).

Here we assessed changes in beliefs, stages of behaviour change and behaviours in the control and treatment groups.

### Beliefs

We found no effect of intervention on beliefs, but did find significant changes in beliefs since the initial survey. Significant changes at two months included the following: (1) Veterinary surgeons were more likely to disagree cats are too small at four months to neuter (63% vs. 43%; LRT _1,232_ = 11.1, p<0.001). (2) Veterinary surgeons were more likely to disagree cats are too young at four months to neuter (69% vs 48%; LRT_1,232_ = 8.7, p = 0.003). (3) Veterinary surgeons were more likely to agree that neutering at four months was beneficial to cat welfare (54% vs 41%; LRT _1,231_ = 4.1, p = 0.04). (4) Veterinary surgeons were more likely to agree that neutering at four months is beneficial to controlling the number of unwanted cats (86% vs 68%; LRT _1,231_ = 10.4, p = 0.001). (5) Veterinary surgeons were more likely to agree that they can make the case to peers around four-month neutering (52% vs. 42%; LRT_1,223_ = 4.4, p = 0.04). There were no significant differences in terms of their agreement that they could make the case to cat owners around four-month neutering, or their agreement that veterinary professionals have a responsibility to encourage four-month neutering.

### Stages of behavioural change

**Fig 2:**
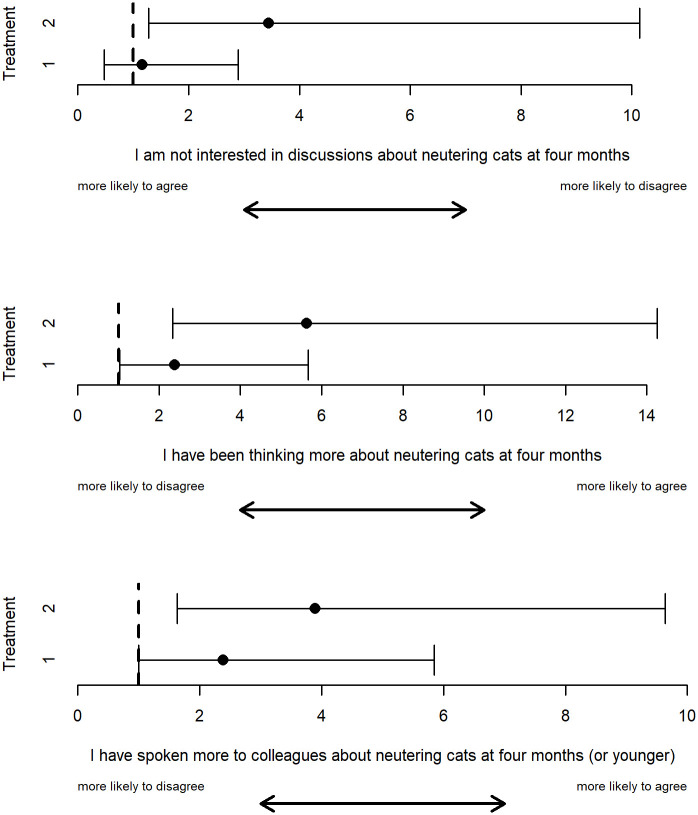
Odds ratios and 95% confidence intervals from the significant ordinal logistic regressions. Treatment 2 was significantly different than the control baseline (dashed line) based on veterinarians self-reported behaviours over the past few months.

We found that veterinary surgeons in the Treatment 2 group were significantly more likely to disagree that they were not interested in discussions around four-month and were significantly more likely to have been thinking more about four-month neutering and have spoken more to colleagues about four month neutering relative to the control group (Table 3, Fig 2). There were no significant differences between Treatment 1 group and the control group (Table 3, Fig 2).

**Table 3:** Agreement with statements regarding intermediate stages of change towards four-month neutering *indicates significant difference between veterinary surgeons in the treatment 1, treatment 2 and control group.

Question	Group	N	Disagree	Neutral	Agree	Ordinal logistic regression statistic
I am not interested in discussions about neutering cats at four months	Control	38	22 (57.9%)	13 (34.2%)	3 (7.9%)	LRT = 6.91 p = 0.03*
	Treatment 1	38	24 (63.2%)	10 (26.3%)	4 (10.5%)	
	Treatment 2	41	34 (82.9%)	6 (14.6%)	1 (2.4%)	
I am not interested in resources about neutering cats at four months	Control	38	25 (65.8%)	9 (23.7%)	4 (10.5%)	LRT = 2.79, p = 0.25
	Treatment 1	38	28 (73.7%)	6 (15.8%)	4 (10.5%)	
	Treatment 2	41	34 (82.9%)	4 (9.8%)	3 (7.3%)	
I have been thinking more about neutering cats at four months	Control	38	14 (36.8%)	13 (34.2%)	11 (29%)	LRT = 15.23, p<0.001
	Treatment 1	37	8 (21.6%)	10 (27%)	19 (51.4%)	
	Treatment 2	41	4 (9.8%)	8 (19.5%)	29 (70.7%)	
I have been actively looking up resources around neutering cats at four months	Control	38	23 (60.5%)	10 (26.3%)	5 (13.2%)	LRT = 2.04, p = 0.36
	Treatment 1	37	20 (54.1%)	12 (32.4%)	5 (13.5%)	
	Treatment 2	39	18 (46.1%)	12 (30.8%)	9 (23.1%)	
I have spoken more to colleagues about neutering cats at four months (or younger)	Control	37	23 (62.2%)	6 (16.2%)	8 (21.6%)	LRT = 9.61, p = 0.008
	Treatment 1	36	13 (36.1%)	11 (30.6%)	12 (33.3%)	
	Treatment 2	41	13 (31.7%)	7 (17.1%)	21 (51.2%)	
I have spoken more to cat owners about four month neutering	Control	36	17 (47.2%)	11 (30.6%)	8 (22.2%)	LRT = 3.5, p = 0.17
	Treatment 1	36	14 (38.9%)	10 (27.8%)	12 (33.3%)	
	Treatment 2	41	14 (34.2%)	8 (19.5%)	19 (46.3%)	
I have started to learn some protocols for four month neutering	Control	36	17 (47.2%)	17 (47.2%)	2 (5.6%)	LRT = 3.2, p = 0.20
	Treatment 1	36	15 (41.7%)	13 (36.1%)	8 (22.2%)	
	Treatment 2	39	16 (41%)	9 (23.1%)	14 (35.9%)	

### Reported behaviour

At the two-month follow-up survey, treatment did not have a significant effect on whether veterinarians reported that they carried out neutering cats at four months, with no significant difference between individuals reporting stopping or starting carrying out the procedure in each group (Table 4).

**Table 4:** Percentages (and numbers) of respondents at each time point that stated they carried out neutering cats at 4 months. Along with the numbers of individuals that stopped carrying out the behaviour and the number of individuals that started. The McNemar test statistic shows the significance of any changes in behaviour.

Group	Pre-intervention (n = 117)	2 months post intervention (n = 117)	Number individuals stopped carrying out	Number individuals started carrying out	McNemar test result
Control	55% (21/38)	39% (15/38)	38% (8/21)	12% (2/17)	Χ^2^ = 2.5, df = 1, p = 0.11
Treatment 1	66% (25/38)	60% (23/38)	16% (4/25)	15% (2/13)	Χ^2^ = 0.17, df = 1, p = 0.68
Treatment 2	54% (22/41)	58% (24/41)	18% (4/22)	31% (6/19)	Χ^2^ = 0.1, df = 1, p = 0.75

However, treatment 2 did have a significant effect on whether veterinarians recommended neutering cats at four months, with significantly more individuals starting the behaviour by the 2-month follow-up survey (Table 5). There was no significant changes within the control or treatment 1 groups.

**Table 5:** Percentages (and numbers) of respondents at each time point that stated they recommend neutering cats at 4 months. Along with the numbers of individuals that stopped recommending and the number of individuals that started. The McNemar test statistic shows the significance of any changes in behaviour *indicates significant difference between the two time points.

Group	Pre-intervention (n = 117)	2 months post intervention (n = 117)	Number individuals stopped recommending	Number individuals started recommending	McNemar test result
Control	21% (8/38)	34% (13/38)	25% (2/8)	23% (7/30)	Χ^2^ = 1.78, df = 1, p = 0.18
Treatment 1	34% (13/38)	42% (16/38)	31% (4/13)	28% (7/25)	Χ^2^ = 0.36, df = 1, p = 0.55
Treatment 2	29% (12/41)	51% (21/41)	17% (2/12)	38% (11/29)	Χ^2^ = 4.9, df = 1, p = 0.03*

### Six-month follow-up

Only respondents that completed the initial, two month and six month follow up surveys were included (n = 85).

### Beliefs

We found no effect of intervention on attitudes, but did find significant changes in attitudes since the initial survey. Significant changes at six months included the following: (1) Veterinary surgeons were more likely to disagree cats are too small at four months to neuter (65% vs. 46%; LRT _1,168_ = 7.44, p = 0.006). (2) Veterinary surgeons were more likely to disagree cats are too young at four months to neuter (68% vs 53%; LRT_1,166_ = 6.2, p = 0.01).

There were no significant differences in terms of their agreement that neutering at four months was beneficial to cat welfare or population control, they could make the case to peers or cat owners around four-month neutering, or their agreement that veterinary professionals have a responsibility to encourage four-month neutering.

### Stages of behavioural change

We found no difference between treatment groups in terms of whether they had contemplated four-month neutering in the last few months, since the previous survey.

### Behaviour

At the six-month follow-up survey, treatment 2 did have a significant effect on whether veterinarians carry out neutering cats at four months, with significantly more individuals starting the behaviour between the pre- and six-month survey (Table 6). There was no significant difference for the control group or treatment 1.

**Table 6:** Percentages (and numbers) of respondents at each time point that stated they carried out neutering cats at 4 months. Along with the numbers of individuals that stopped carrying out the behaviour and the number of individuals that started between the pre- and six-month survey. The McNemar test statistic shows the significance of any changes in behaviour *indicates significant difference between the two time points.

Group	Pre-intervention (n = 85)	2 months post intervention (n = 85)	6 months post intervention (n = 85)	Number individuals stopped carrying out	Number individuals started carrying out	McNemar test result
Control	46% (12/26)	38% (10/26)	50% (13/26)	33% (4/12)	36% (5/14)	Χ^2^ = 0, df = 1, p = 1
Treatment 1	68% (19/28)	68% (19/28)	75% (21/28)	5% (1/19)	33% (3/9)	Χ^2^ = 0.25, df = 1, p = 0.62
Treatment 2	55% (17/31)	58% (18/31)	81% (25/31)	6% (1/17)	64% (9/14)	Χ^2^ = 4.9, df = 1, p = 0.03*

At the six-month follow-up survey, despite an increase in veterinarians in the treatment 2 group recommending neutering cats at four months, this effect was insignificant (Table 7).

**Table 7:** Percentages (and numbers) of respondents at each time point that stated they recommended neutering cats at 4 months. Along with the numbers of individuals that stopped recommending and the number of individuals that started between the pre- and six-month survey. The McNemar test statistic shows the significance of any changes in behaviour.

Group	Pre-intervention (n = 85)	2 months post intervention (n = 85)	6 months post intervention (n = 85)	Number individuals stopped recommending	Number individuals started recommending	McNemar test result
Control	23% (6/26)	38% (10/26)	27% (7/26)	17% (1/6)	10% (2/20)	Χ^2^ = 0, df = 1, p = 1
Treatment 1	39% (11/28)	50% (14/28)	53% (15/28)	18% (2/11)	35% (6/17)	Χ^2^ = 1.1, df = 1, p = 0.28
Treatment 2	32% (10/31)	48% (15/31)	52% (16/31)	10% (1/10)	33% (7/21)	Χ^2^ = 3.1, df = 1, p = 0.07

## Discussion

Here we describe use of a video intervention to prompt voluntary behavioural change towards four-month neutering of owned cats. In so doing, we have outlined the value of a one-off intervention to bring about behaviours that align with the four-month neutering recommendation, while preserving clinical autonomy. Our control conditions were purposefully designed, controlling not just for external influences, but also by having a written summary of evidence group, we controlled for information exposure to disentangle the importance of delivery from a peer and authoritative source from information alone. Our findings demonstrate the efficacy of the social aspect of the intervention in demonstrating behaviour change over a period of six months. These data-led insights outline an approach that could increase the consistency in recommending four month neutering of pet cats, both in the UK and in other international contexts where barriers to prepubertal neutering are similar.

In the video intervention group, self-reports of thinking about, and speaking to colleagues about, four-month neutering significantly increased in the two months following the intervention. These medium-term changes in openness to discussions and associated additional thought processes may be the mechanism behind the observed longer-term intervention effects. Indeed, we find veterinary surgeons that watched the video intervention were more likely to be recommending and carrying out neutering of four-month old owned cats, at two months and six months following the intervention respectively. In contrast, although veterinary surgeons that received the written summary of evidence found it to be a more helpful, knowledge-based resource immediately following delivery, we did not find the same significant changes in behaviour in the longer-term. This finding indicates it is not the evidence base alone but may be the demonstration of the evidence by peers as the influential factor.

In terms of our COM-B framework, these results suggest the video intervention provided the psychological capability because it demonstrated the evidence-base around the behaviour [46], the social opportunity in highlighting practice norms and the motivation in terms of the confidence to reflect on their neutering practices, have subsequent conversations with colleagues and perform the behaviour. It will have also potentially relieved any concerns they may have around outcome expectancies in hearing the positive outcomes, as discussed in the video. Social influences are equally found to be important for other self-regulatory professions such as physicians [47], who similarly work in time- and resource-pressured environments where decision-making may become subject to personal cognitive biases, routines and habits. Indeed, given the behaviours of veterinary surgeons reflect their experience within the practice [15], the video potentially increased the observability of behaviour that they may not see in their day-to-day clinic [48]. These results suggest that in situations where clinical behaviours may be entrenched and reflective of historical norms, that social influences can increase the appeal of a behaviour through a variety of mechanisms and result in a voluntary behaviour shift.

We found that none of the veterinary surgeons that accessed the video intervention re-watched it. This is perhaps unsurprising as the video intervention is not solely an educational resource, and further highlights its benefits largely lie in its social and modelling aspects. The intervention was one-off and brief and did nothing to restrict clinicians’ choice of treatment, therefore making a negligible change to the veterinary surgeons environment. Similarly, in situations where education alone is not effective in changing care, interventions that gently nudge clinician decision making while preserving autonomy have been found to be effective for other health providers [49]. Given the success of the video as a one-off intervention, its brevity lends itself to be shared within the veterinary profession as additions to lectures, training and online conference platforms. Although, veterinary students were not the focus of this study, the video may be beneficial to them along with practical experience, exposing students to four-month neutering for client-owned cats potentially pre-empting within-practice experience, which can be a key influencer of behaviours towards four-month neutering [15]. Additionally, despite limited research on how prepubertal neutering is taught across UK universities, it is often considered within the remit of shelter medicine, where prepubertal neutering (often younger than four months) is considered safe and appropriate [14]. This approach may preclude some students from gaining an understanding of four-month neutering and inadvertently reinforce the idea that it is not applicable for client-owned cats, further highlighting the value of providing information on prepubertal neutering for client-owned cats during these training years.

The written summary of evidence was viewed as a significantly more helpful and relevant resource compared to the video intervention. Additionally, unlike the video intervention it was accessed on more than one occasion by 13% of the group. Despite this, we do not find equivalent changes in behaviour for veterinary surgeons that received the written information. This is perhaps surprising given many veterinary surgeons cite a lack of evidence as a barrier to carrying out the behaviour [15]. Similar results have been found in other studies of health science where socially motivated interventions are superior to education alone [50]. These findings highlight the pitfalls of relying on feedback evaluation alone, which is unable to detect normative influences [36].

Interestingly, we found a general improvement in attitudes through time across all groups including the control group, whereby veterinary surgeons had more positive beliefs on the neutering of four-month old cats at both two and six months. It is not clear whether this temporal trend is due to engagement in this research or reflective of changes occurring across the veterinary profession in the UK. However, despite positive changes in beliefs across all groups we did not see equivalent changes in behaviours, highlighting the importance of social influences to instigate behaviour change within six months. We cannot discount however, that behaviours would change across all groups eventually and the intervention merely expedited this process.

The results reported here demonstrate not only the effectiveness of the video intervention but also the underpinning mechanism of reflection from the intervention participants that lead them to increased openness towards discussions around four-month neutering. Although promising, these results should be interpreted with some consideration of the limitations of the study, such as the reduced sample sizes at the latter end of the study and the potential for response bias due to the self-report nature of the data.

Whilst, respondents in the treatment groups were required to confirm they had accessed and viewed the content, this was a self-reported measure. Therefore, we cannot discount that the video format lends itself to being more accessible and more likely to have been viewed in full and this may have influenced the change in behaviours that were reported. However, we found that the video intervention was not reported as being accessed again and was not viewed as favourably indicating that the delivery format may have limited impact on our findings. Nevertheless, it is unknown the extent to which the video format alone impacted the behaviour change we observe.

Additionally, whilst our questioning focuses on behaviours despite practice policies, the positive changes we observe may or may not be implemented due to intervening practice policies. Therefore, our study does not account for perceived behavioural control. It is unclear the content of discussions with colleagues around four-month neutering and whether these have the potential to enhance motivation to have an effect on practice policy changes resulting in practice-level support. Practice-based neutering policies are important indicators of four-month neutering [15] with further research needed to understand how best to support veterinarians to make within practice policy changes. We also recognise that the video intervention prompted the behaviour of some but not all veterinary surgeons, and further training or information may be needed for some individuals. Nevertheless, these results are encouraging and offer an additional approach to better support veterinary surgeons.

To conclude, in this trial we outlined how a one-off video intervention significantly changed the behaviours of veterinary surgeons towards neutering cats at 4 months, likely mediated by the social influences of the intervention prompting the opportunity to reflect and discuss four-month neutering with colleagues. In doing so, we highlight more generally how insights from behavioural science can be used to develop effective interventions within the field of veterinary medicine, which may be applicable in other contexts where clinical practises within the profession are inconsistent. The intervention itself is brief, targeted and does not influence the autonomy of veterinary surgeons to make decisions. Instead, it prompts the opportunity to reflect upon their own practices both individually and with their colleagues and we suggest that there should be more opportunities to do so within the profession to improve the consistency in care, to approach that of the recommended neutering standards.

## Supporting information

S1 Dataset Participant responses to Likert scale questioning. (XLSX)

## References

[1] Mcdonald JL, Skillings E. Human influences shape the first spatially explicit national estimate of urban unowned cat abundance. Sci Rep. 2021; 1–12. doi: 10.1038/s41598-020-79139-8 34711904 PMC8553937

[2] Levy JK, Crawford PC. Humane strategies for controlling feral cat populations. J Am Vet Med Assoc. 2004;225: 1354–1360. doi: 10.2460/javma.2004.225.1354 15552308

[3] Jessup DA. The welfare of feral cats and wildlife. J Am Vet Med Assoc. 2004;225: 1377–1383. doi: 10.2460/javma.2004.225.1377 15552312

[4] Lowe S, Browne M, Boudjelas S, De Poorter M. 100 of the world’s worst invasive alien species: a selection from the global invasive species database. Invasive Species Specialist Group Auckland; 2000.

[5] Clark CCA, Gruffydd-Jones T, Murray JK. Number of cats and dogs in UK welfare organisations. Vet Rec. 2012;170: 493. doi: 10.1136/vr.100524 22589036

[6] Stavisky J, Brennan ML, Downes M, Dean R. Demographics and economic burden of un-owned cats and dogs in the UK: results of a 2010 census Demographics and economic burden of un-owned cats and dogs in the UK: results of a 2010 census. BMC Vet Res. 2012;8. 10.1186/1746-6148-8-163 PMC3514250 22974242

[7] McDonald JL, Hodgson D. Counting Cats: The integration of expert and citizen science data for unbiased inference of population abundance. Ecol Evol. 2021;11: 4325–4338. doi: 10.1002/ece3.7330 33976813 PMC8093703

[8] PDSA. PDSA Animal wellbeing report (PAW) 2020. 2020. https://www.pdsa.org.uk/media/10540/pdsa-paw-report-2020.pdf.

[9] Cats Protection. CATS (Cats and Their Stats) 2020 UK. 2020. https://www.cats.org.uk/assets/cats-report/Cats_Report_2020.pdf.

[10] Joyce A, Yates D. Help stop teenage pregnancy!. Early-age neutering in cats. J Feline Med Surg. 2011;13: 3–10. doi: 10.1016/j.jfms.2010.11.005 21215944 PMC10845413

[11] The cat group. Policy Statement 1: Timing of neutering. [cited 22 Jun 2021]. http://www.thecatgroup.org.uk/policy_statements/neut.html.

[12] The Cat Population Control Group. KiND: Kitten Neutering Database. [cited 22 Jun 2021]. http://www.kind.cats.org.uk/.

[13] BSAVA. Neutering of Dogs, Cats, Rabbits and Ferrets. 2019 [cited 22 Jun 2021]. https://www.bsava.com/Resources/Veterinary-resources/Position-statements/Neutering.

[14] British Veterinary Association. Policy statement. Neutering of cats and dogs. 2019 [cited 22 Jun 2021]. https://www.bva.co.uk/media/1167/neutering-cats-dogs-policy-print.pdf.

[15] McDonald J, Clements J. Contrasting practices and opinions of UK-based veterinary surgeons around neutering cats at four months old. Vet Rec. 2020;187. doi: 10.1136/vr.105887 32764034 PMC7606499

[16] Mazeau L, Wylie C, Boland L, Beatty JA. A shift towards early-age desexing of cats under veterinary care in Australia. Sci Rep. 2021;11: 1–9. doi: 10.1038/s41598-020-79139-8 33462250 PMC7813850

[17] Welsh CP, Gruffydd-Jones TJ, Murray JK. Paper: The neuter status of cats at four and six months of age is strongly associated with the owners’ intended age of neutering. Vet Rec. 2013;172: 578. doi: 10.1136/vr.101362 23605077

[18] Welsh CP, Gruffydd-Jones TJ, Roberts MA, Murray JK. Poor owner knowledge of feline reproduction contributes to the high proportion of accidental litters born to UK pet cats. Vet Rec. 2014;174: 118. doi: 10.1136/vr.101909 24343905

[19] Wongsaengchan C, McKeegan DEF, Wongsaengchan C, McKeegan DEF. The Views of the UK Public Towards Routine Neutering of Dogs and Cats. Anim 2019, Vol 9, Page 138. 2019;9: 138. doi: 10.3390/ani9040138 30986979 PMC6523704

[20] Gagnon AC, Langlade C, Buff S, Rosset E. A retrospective internet-based survey of French cat breeders about early-age neutering. J Feline Med Surg. 2019; 1–7. doi: 10.1177/1098612X19858800 31264521 PMC10814338

[21] Farnworth MJ, Adams NJ, Seksel K, Waran NK, Beausoleil NJ, Stafford KJ. Veterinary attitudes towards pre-pubertal gonadectomy of cats: a comparison of samples from New Zealand, Australia and the United Kingdom Veterinary attitudes towards pre-pubertal gonadectomy of cats: a comparison of samples from New Zealand, Australi. N Z Vet J. 2013;61: 226–233. doi: 10.1080/00480169.2012.738591 23227915

[22] Paterson MBA, O’donoghue M, Jamieson P, Morton JM. The cat desexing policies and activities of private veterinary practices in Queensland. Animals. 2020;10: 1–15. doi: 10.3390/ani10050841 32414142 PMC7278410

[23] Gagnon AC, Langlade C, Rosset E, Buff S. French veterinarians’ opinions and practices regarding early neutering of cats: A convenience sampling survey interpreted in an international context. Vet Rec. 2020;187: E120. doi: 10.1136/vr.105944 32978276

[24] Spain CV, Scarlett JM, Cully SM. When to neuter dogs and cats: A survey of New York state veterinarians’ practices and beliefs. J Am Anim Hosp Assoc. 2002;38: 482–488. doi: 10.5326/0380482 12220034

[25] Orr B, Jones B. A Survey of Veterinarian Attitudes Toward Prepubertal Desexing of Dogs and Cats in the Australian Capital Territory. Front Vet Sci. 2019;6: 1–7. doi: 10.3389/fvets.2019.00001 31497606 PMC6712071

[26] Roberts ML, Beatty JA, Dhand NK, Barrs VR. Effect of age and surgical approach on perioperative wound complication following ovariohysterectomy in shelter-housed cats in Australia. J Feline Med Surg Open Reports. 2015;1: 205511691561335. doi: 10.1177/2055116915613358 28491391 PMC5362017

[27] Joyce A, Yates D. Help stop teenage pregnancy!. Early-age neutering in cats. J Feline Med Surg. 2011;13: 3–10. doi: 10.1016/j.jfms.2010.11.005 21215944 PMC10845413

[28] Howe LM, Slater MR, Boothe HW, Hobson HP, Fossum TW, Spann AC. Long-term outcome of gonadectomy performed at an early age or traditional age in cats. J Am Vet Med Assoc. 2000;217: 1661–1665. doi: 10.2460/javma.2000.217.1661 11110455

[29] Howe LM. Short-term results and complications of prepubertal gonadectomy in cats and dogs. J Am Vet Med Assoc. 1997;211: 57–62. 9215412

[30] Murray JK, Skillings E, Gruffydd-Jones TJ. Opinions of veterinarians about the age at which kittens should be neutered. Vet Rec. 2008;163: 381–385. doi: 10.1136/vr.163.13.381 18820325

[31] Jackson C, Eliasson L, Barber N, Weinman J. Applying COM-B to medication adherence. Bull Eur Heal Psychol Soc. 2014;16: 7–17. Available: http://www.ehps.net/ehp/issues/2014/v16iss1February2014/16_1_EHP_Februay2014Jacksonetal.pdf.

[32] Michie S, van Stralen MM, West R. The behaviour change wheel: A new method for characterising and designing behaviour change interventions. Implement Sci. 2011;6. doi: 10.1186/1748-5908-6-42 21513547 PMC3096582

[33] Michie S, Atkins L, West R. The behaviour change wheel: a guide to designing interventions. 2014. Gt Britain Silverback Publ. 2015.

[34] Cane J, O’Connor D, Michie S. Validation of the theoretical domains framework for use in behaviour change and implementation research. Implement Sci. 2012;7: 1–17. doi: 10.1186/1748-5908-7-37 22530986 PMC3483008

[35] Honda K, Gorin SS. A model of stage of change to recommend colonoscopy among urban primary care physicians. Heal Psychol. 2006;25: 65. 10.1037/0278-6133.25.1.65 16448299

[36] Nolan JM, Schultz PW, Cialdini RB, Goldstein NJ, Griskevicius V. Normative social influence is underdetected. Personal Soc Psychol Bull. 2008;34: 913–923. doi: 10.1177/0146167208316691 18550863

[37] Inglis M, Mejia-Ramos JP. The effect of authority on the persuasiveness of mathematical arguments. Cogn Instr. 2009;27: 25–50.

[38] McGinnies E, Ward CD. Better liked than right: Trustworthiness and expertise as factors in credibility. Personal Soc Psychol Bull. 1980;6: 467–472.

[39] Carpenter CR, Sherbino J. How does an “opinion leader” influence my practice? Can J Emerg Med. 2010;12: 431–434. doi: 10.1017/s1481803500012586 20880436 PMC3217217

[40] Ajzen I. From intentions to actions: A theory of planned behavior. Action control. Springer; 1985. pp. 11–39.

[41] Bandura A. Social cognitive theory of self-regulation. Organ Behav Hum Decis Process. 1991;50: 248–287.

[42] Vogt F, Hall S, Hankins M, Marteau TM. Evaluating three theory-based interventions to increase physicians’ recommendations of smoking cessation services. Heal Psychol. 2009;28: 174. doi: 10.1037/a0013783 19290709

[43] R Core Team. R: A language and environment for statistical computing. R Foundation for Statistical Computing, Vienna, Austria. 2020.

[44] Ripley B, Venables B, Bates DM, Hornik K, Gebhardt A, Firth D, et al. Package ‘mass.’ Cran r. 2013;538: 113–120.

[45] Fox J, Weisberg S. Multivariate linear models in R. An R Companion to Appl Regression Los Angeles Thousand Oaks. 2011.

[46] Chater A, Milton S, Green J, Gilworth G, Roposch A. Understanding physician behaviour in the 6–8 weeks hip check in primary care: A qualitative study using the COM-B. BMJ Open. 2021;11: 1–7. doi: 10.1136/bmjopen-2020-044114 33741671 PMC7986785

[47] Ryu S, Ho SH, Han I. Knowledge sharing behavior of physicians in hospitals. Expert Syst Appl. 2003;25: 113–122. doi: 10.1016/S0957-4174(03)00011-3

[48] Prentice DA. Intervening to change social norms: When does it work? Soc Res (New York). 2018;85: 115–139.

[49] Lamprell K, Tran Y, Arnolda G, Braithwaite J. Nudging clinicians: A systematic scoping review of the literature. J Eval Clin Pract. 2021;27: 175–192. doi: 10.1111/jep.13401 32342613

[50] Meeker D, Linder JA, Fox CR, Friedberg MW, Persell SD, Goldstein NJ, et al. Effect of behavioral interventions on inappropriate antibiotic prescribing among primary care practices a randomized clinical trial. JAMA—J Am Med Assoc. 2016;315: 562–570. doi: 10.1001/jama.2016.0275 26864410 PMC6689234

